# In uncharted territory “together each achieves more”: a United Nations interagency collaboration for continuity of maternal and newborn health services during the coronavirus pandemic in the Eastern and Southern Africa region

**DOI:** 10.3389/frhs.2023.1230414

**Published:** 2023-08-31

**Authors:** Anne-Marie Bergh, Fatima Gohar, Nancy A. Kidula, Muna Abdullah

**Affiliations:** ^1^ Research Centre for Maternal, Fetal, Newborn and Child Health Care Strategies, Faculty of Health Sciences, University of Pretoria, Pretoria, South Africa; ^2^ South African Medical Research Council Unit for Maternal and Infant Health Care Strategies, Faculty of Health Sciences, University of Pretoria, Pretoria, South Africa; ^3^ UNICEF Eastern and Southern Africa Regional Office, Nairobi, Kenya; ^4^ Reproductive and Women’s Health, WHO AFRO Intercountry Support Team for East and Southern Africa, Harare, Zimbabwe; ^5^ UNFPA East and Southern Africa Regional Office, Johannesburg, South Africa

**Keywords:** dimensions of collaboration, COVID-19 pandemic response, e-learning, quality of care, skills development

## Abstract

The frangible collaboration between three United Nations agencies (UNICEF, UNFPA and WHO) in the Eastern and Southern Africa Region was strengthened by the outbreak of the coronavirus pandemic. The aim was to combine existing resources and expertise to support countries to respond to the pandemic more effectively and efficiently regarding the provision of maternal and newborn health services. Three kinds of activities were conducted: 15 webinars on a variety of topics and issues impacted by the pandemic; virtual training on maternal and perinatal death surveillance and response as well as on quality improvement; and the development of online e-learning modules for continuous professional development. Key dimensions of the collaboration included: a common vision; commitment to the process; dialogue; building relationships and trust; communication and information sharing; sharing of technical and financial resources and expertise; mobilization of additional resources; celebration of intermediate outcomes; facilitative leadership; and institutional design. Start-up lessons revolved around shared risk taking, while retaining agency autonomy. Collaboration lessons included forming a “united front”, harnessing technology to accelerate results, and mitigating adverse structural and contextual factors. There are widespread perceptions that collaborative initiatives tend to yield minimum results in terms of increased efficiency or effectiveness. This particular collaborative effort demonstrated elements of feasibility, value addition, synergy, cost effectiveness and demonstrable results where UN agencies delivered as one. The emergency in healthcare as a ripple effect of the coronavirus pandemic has caused a rethink of collaboration models and levels of engagement.

## Introduction

The concept of collaboration among diverse partners, disciplines and professional groups has gained significant acceptance in recent years. A well-coordinated partnership and collaborative effort are critical in delivering better results towards a shared goal. The H6 is a global partnership between six United Nations (UN) agencies collaborating with various other development agencies. Their shared goal is to advance women's, children's and adolescents' health in high-burden countries and act as a technical support arm for the Every Woman, Every Child movement. The H6 partnership also provides a niche for coordinated and coherent contextually defined technical support in the UN Eastern and Southern Africa (ESA) Region, with all countries, except Botswana and Somalia, involved in some form of H6 collaboration in 2020. Regional coordination aims to increase volume and quality of technical support, enhance advocacy and policy engagement, minimize duplication and mobilize more resources for sexual, reproductive, maternal, newborn, child and adolescent health (SRMNCAH) in the region (1, 2).

**Figure 1:**
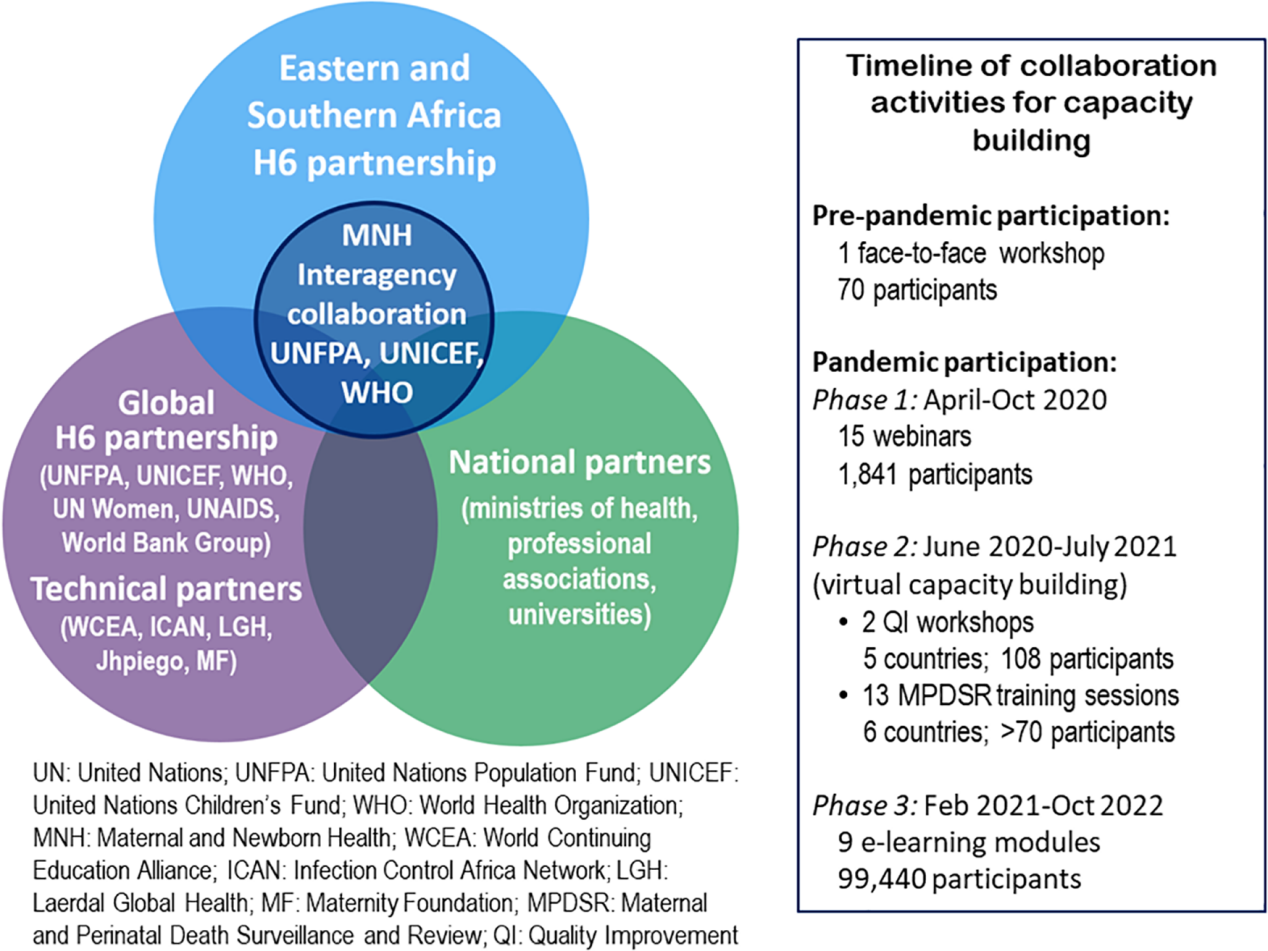
The MNH interagency collaboration within the bigger picture.

Within the regional H6 partnership, the UN Population Fund (UNFPA), the UN Children's Fund (UNICEF) and the World Health Organization (WHO) operating in the 23 countries of the ESA Region mutually acknowledged overlaps, duplication, and inadequate and fragmented resources for maternal and newborn health (MNH). In 2018 they embarked on strengthening the interagency collaboration to harness the collective strengths of the three agencies. Figure 1 is a graphic depiction of this collaboration in relation to both the global and regional H6 partnership and other partners involved in the collaboration. The collaboration acquired new urgency and meaning with the advent of the coronavirus pandemic in 2020. Essential MNH services in ESA, as in other parts of the world, were extremely constrained during the early phases of the outbreak. Pregnant women and their newborns were disproportionately affected by the pandemic and there was a real threat of reversal of recent improvements in SRMNCAH care.

It was vital to use the limited resources available in the interrelated MNH program areas more effectively in an integrated, comprehensive and results-driven approach. With a few exceptions, the key country actors were MNH focal points in their respective agencies.

## Our collaborative approach

This article is the outcome of consulting internal reports of the three agencies and reflective discussions between the four authors, which included summarising all the activities that took place during this collaboration and providing an interpretive lens on the collaboration as a whole. We wanted to demonstrate how our joint work was a more efficient and cost-effective response to the pandemic than working as individual agencies. The paper also highlights the levels and instruments of collaboration that fostered cooperation and describes experiences regarding the collaboration process and the lessons learnt from the nuts and bolts of a joint implementation.

### Why did we collaborate?

The coronavirus pandemic and a “prehistory of cooperation” were the “starting conditions” (3) that triggered our efforts to strengthen collaboration. There was an urge to build on existing collaboration to mitigate the risk of disruption of MNH services. The challenge was too urgent and overwhelming to be tackled by individual efforts and the following aims emerged from the situation:

1. Combine wider skills and resources to tackle the threatened disruption of MNH services that might have cost the lives of women and newborns;
2. respond to country needs in a coherent and holistic manner and deliver quality technical assistance;
3. take action faster, using the various mechanisms available within each agency; and
4. model a joint UN team at regional office level with the aspiration and expectation that this approach of a unified UN team would also be mirrored at country-office level.

COVID-19 was a novel disease at the time and information on how to manage pregnancy and childbirth within the pandemic context was limited. Misconceptions on COVID-19 infection in Africans and in pregnant women and newborns were rife. In the early days of the pandemic MNH services were not prioritized and reports of maternity units being repurposed as COVID centres and of maternity staff being reassigned to COVID wards abounded. In addition, the institution of public health measures to combat the virus led to media reports—the only source of information at the start of pandemic—of delays in pregnant women reaching health facilities, of transporters refusing to take women to hospital at night due to curfews, and inevitably of deaths of pregnant women (4–8).

The first case of COVID-19 in the ESA Region was reported in March 2020 in South Africa. Countries urgently requested guidance from the UN ESA team since the global guidance at that time did not include specific guidance on pregnancy and childbirth. The first joint webinar was held on 9 April to discuss the regional situation regarding COVID-19. The emphasis fell on the MNH situation in South Africa, where the lockdown measures had been implemented a few days before. By 17 April, joint technical guidance on the continuity of MNH services during COVID-19 had been developed and shared, later to be updated as global guidance became available. The joint ESA team decided to work together to avoid potentially fragmented operational guidance and technical briefs that would have led to inefficient service implementation. The aim was to provide easy, practical, step-by-step guidance to inform the provision of MNH services in the ESA countries (9).

### What form did collaboration take?

When restrictive pandemic lockdown measures came into effect, in-person technical support to countries was suspended due to prevailing travel restrictions. With global evidence generation advancing rapidly, it was important to obtain synthesized evidence-based guidance from the global secretariat without delay. The initial task of the collaboration was to disseminate operational guidance on the provision of MNH services during the pandemic. Face-to-face training had to be replaced by virtual or hybrid models of blended learning, where country teams implemented the interventions at selected learning sites after receiving some didactic sessions. This need for guidance led to the following joint activities by the three agencies to support countries in three phases (see also Figure 1): (i) an initial series of webinars held between April and October 2020; (ii) virtual capacity building in quality of care (June to October 2020) and maternal and perinatal death surveillance and response (MPDSR) (November 2020 to July 2021); and (iii) the development and testing of online e-learning modules as part of continuous professional development (CPD) during the remaining months of 2020 and in 2021. These activities reached more participants than traditional face-to-face activities.

**Phase 1. Webinars** (10). An initial series of 15 webinars was held with a diverse group of 1,841 participants. Attendance ranged between 85 and 164 participants per webinar (median 124 participants) who included officials from ministries of health (and social services), UN agencies at country level, development agencies (e.g., Save the Children, PATH), professional organizations, universities, and country-level non-governmental organizations. The organization of each webinar was a joint responsibility, with each agency undertaking specific tasks (see Supplementary Table S1 in the Supplementary File). The regional focal persons of the collaboration agreed on key topics, deliberated which agency had the comparative technical advantage to present the topic, and analysed the different organizations to see which administrative and logistical measures could more easily be undertaken by each agency. For example, UNICEF was responsible for setting up the zoom facilities and sending out invitations, which were then further distributed through the other two agencies. The topics included the following: COVID-19 themes related to various aspects of service delivery and care across the MNH continuum; country experiences with the pandemic; guidance on clinical care during pregnancy, childbirth and immediately after birth in the context of COVID-19 (including quality care for small and sick newborns, who are the most vulnerable when the health system is strained); infection prevention and control (including personal protective equipment); gender-based violence (which showed high increases in some countries during the early phase of the pandemic); and MPDSR.

The webinars were inclusive and participatory. From the beginning feedback was collected from participants on the usefulness of the sessions, and actions taken at country level as a result of the webinars were checked. Participants were also asked to indicate specific areas on which they needed a webinar or more information and guidance. These requests were then used to plan for subsequent webinars and training events with a view to developing the national capacities around unified guidelines and technical support, and to creating a place for cross-learning and experience-sharing among member states.

**Phase 2. Virtual capacity building.** In addition to the webinars, the collaboration launched a regional virtual training program in MPDSR under the 2gether 4 SRHR program. The aim was to support countries to accurately document the magnitude of maternal and perinatal deaths, and identify, classify, and report the underlying causes of death so as to inform correct management and systems strengthening. This was also critical for identifying maternal and perinatal deaths associated with COVID-19. Over 70 participants from Kenya, Namibia, Rwanda, Uganda, Zambia and Zimbabwe took part in the 13 consecutive sessions. Topics included the following: identification and notification of maternal deaths occurring in health facilities; medical certification of cause of death; international classification of cause of death; linking MPDSR with Integrated Disease Surveillance and Response (IDSR) and health management information systems (HMIS); and report-back from countries.

There were also country initiatives to strengthen quality of care in MNH. One example was the virtual capacity-building workshops in quality improvement for multidisciplinary teams and coaches organized by the regional MNH collaboration and the agencies' country offices in coordination with ministries of health in Kenya and Ethiopia (11). The main goal of these workshops was to prepare and equip a pool of resource personnel for effective implementation of the quality improvement agenda at the national, regional, district and health facility levels. Observers from Ethiopia, Tanzania, Malawi, and Namibia attended the Kenya workshop with the aim of generating interest and replicating similar workshops in their respective countries.

**Phase 3. E-learning.** With the increasing demand for practical information and the high attendance of webinars even beyond ESA, it became imperative to transfer existing modules onto an e-learning platform. This was intended to increase access to a wider audience, while allowing health workers to obtain CPD points.

Nine e-learning modules were developed and hosted in the World Continuing Education Alliance (WCEA) platform. The modules cover the WHO Labour Guide (1 module), Maternal Death Surveillance and Response (MDSR) (4 modules) and Perinatal Death Surveillance and Response (PDSR) (4 modules) (see Supplementary Table S2 in the Supplementary File). All modules are available in English, and some have also been translated into French and Portuguese. Between February 2021 and October 2022, 216,487 course modules were taken by 99,440 learners, with about half of the participants residing in the ESA Region and the other half in other regions.

### Key dimensions of the collaboration

A key feature of this collaboration was the ability to leverage the comparative advantage of each agency. The regional offices of the agencies are geographically dispersed, each with their own varied governing structures, administrative procedures, and program niches. WHO is mandated to avail and promptly share the most up-to-date evidence-based global guidance; UNICEF and UNFPA support strengthening the capacity of national health systems and processes so that no child or mother is left behind. The synergy between the interagency partners was further enhanced by the WHO's ability to harness area experts to address the technical elements of the webinars and training, with UNFPA having the edge in monitoring and evaluation, data analytics and oversight of the virtual training platforms. UNICEF had the edge in perinatal and child health issues and quality of care, and took the lead in coordination. Furthermore, the members of the regional collaboration were committed to transparency in working together towards a common goal.

Negotiating the “ground rules” for collaboration (12) was facilitated by the fact that all three agencies were part of the UN institutional family, each with its own extensive network of other partners. The collective commitment of the three agencies to ensuring continuity of MNH services during the pandemic steered the collaboration in three main areas of the H6 model: technical support, convening role, and advocacy (2). The technical area included availing up-to-date evidence-based guidance to facilitate the continuity of MNH services during the pandemic, monitoring and evaluation of the provision of services, monitoring of program data, and the periodic dissemination of reports. Convening entailed setting up a technical advisory group, coordinating the webinars, establishing the e-learning platform, and convening other collaborative meetings. Advocacy was focused on guiding national governments to prioritize MNH services within the pandemic agenda to ensure that COVID-19 measures integrated actions to enhance the continuity of MNH services. There was also a focus on promoting collaboration on digital health for MNH with regional communities such as the Southern African Development Community (SADC), the East African Community (EAC), and the African Union Commission (AUC).

We used and adapted Ansell and Gash's dimensions of a collaborative process (12) to organise information and observations of how our collaboration functioned. The dimensions include: a common vision; commitment to the process; dialogue; building relationships and trust; communication and information sharing; sharing of technical and financial resources and expertise; mobilization of additional resources; celebration of intermediate outcomes and “small wins”; facilitative leadership; and institutional design. Table 1 contains a more detailed description of each dimension.

**Table 1:** Dimensions of the collaborative process.

Dimension	Description
Common vision and a shared understanding of the fundamental objectives of the collaboration	• Common realization that more needed to be done to coordinate efforts, assist the region in improving MNCH services and avoid duplication of efforts. • Shared vision to: improve the lives of women and children in the region throughout the coronavirus pandemic; improve the delivery of respectful maternal, newborn and sexual and reproductive health services; and protect frontline health workers. • Holistic approach needed in line with the Every Woman Every Child movement that includes the UN as a whole, governments as a whole and society as a whole.
Commitment to the process	• Commitment of the three regional focal persons. • All three agencies committed people, time and resources to the process.
Dialogue	• Switching from face-to-face modes of dialogue to virtual meetings and events required adaptation of some processes. • Individual collaboration partners held bilateral dialogues with specific external partners who were subsequently brought on board with certain activities (e.g. the work of UNFPA with WCEA and midwifery associations; UNICEF with UP and ICAN; WHO with Africa CDC and obstetrics and gynaecology associations). (See also Figure 1.) • Dialogue with various teams in each agency (e.g. humanitarian response team, gender team, data team). • Ongoing virtual dialogue between the regional and country collaborations. • In-country dialogues with the different departments of the ministries of health (e.g. Safe Motherhood or equivalent task team, health information system, COVID case management team, COVID response coordination team).
Investing in building relationships and trust	• Early communication to ensure that all partners remained enthusiastic about the relationship—nurturing relationships with external partners and funders. • The prior, less formal arrangement between the three agencies facilitated the development of confidence and the setting of shared standards. • Country offices of the three agencies encouraged to strengthen similar collaborative processes at country level. • The link between H6 at global and country levels strengthened by focusing on the unique contexts surrounding women, children and adolescents in the ESA Region.
Effective communication and information sharing	• Actively sought new guidance (e.g. by subscribing to notifications, joining global task teams, regular reaching out to headquarter activities on COVID-19 responses). • Shared global guidance issued by each agency at international and regional level through immediate dissemination to countries. All updates and recommendations shared almost as soon as they appeared. • Collected, collated and shared national guidelines on COVID-19 responses; national tools (e.g. rapid health facility assessment on readiness of health facilities to respond to COVID-19); information produced at country level (e.g. reports of incidences of blocked access to MNH services); and on-going global research for countries to prepare in advance [e.g. estimation of impact of the pandemic on MNH (13)]. • Ensured the technical accuracy of the draft training materials and modules. • Used knowledge-sharing platforms and communication avenues to publicize webinars and inform member countries about them and share recordings afterwards; also used e-learning modules as they became available. • Leveraged networks to increase enrolment and training attendance.
Sharing of technical and financial resources and expertise, and mobilization of additional resources	• Collaboration aimed at increased volume and quality of technical support, minimization of duplication and mobilization of more resources for RMNCAH in the region through revised work plans to allocate additional resources to activities related to this effort; as well as resource mobilization from existing funds (e.g. the 2gether 4 SRHR joint program) to develop a joint work plan and allocate resources for each agency and beyond (UNAIDS included). • Mobilization of additional resources for capacity building by developing and presenting relevant capacity building activities, and contracting a technology agency to avail the WCEA virtual learning platform. • Joint monitoring and assessments of the impact of COVID-19 on SRMNCAH services and countries’ progress with pandemic response coordination (e.g. a national plan to ensure continuity of services; tracking of SRMNCAH services heavily impacted by COVID-19).
Celebration of intermediate outcomes and “small wins” (12)	• Examples of joint support: - Eritrea Ministry of Health: development of their RMNCAHN strategic plan at regional office and country-office level; and - Zanzibar: capacity building in MPDSR. • By September 2020 all countries in the ESA region (except Seychelles) had disseminated key interim operational guidance documents generated at global and regional level, including the Continuity of Minimum Essential Maternal and Newborn Health Services in the Context of COVID-19. Joint Interim Technical Reference Note produced by this collaboration (9). • Intermediate outcomes are also described in the text narrative of this paper, e.g. the evolution of the 15 webinars across time and the development of e-learning modules (See also Supplementary Tables S1 and S2 in the Supplementary File). • Participant initiatives emanating from the webinars: - prioritization of services during the various phases of COVID-19 in individual countries; - Botswana: inclusion of joint regional guidelines in national service continuity plan; - bilateral conversations between individual countries regarding tools, training, national coordination mechanisms and local solutions for IPC; and - WhatsApp groups and use of other online platforms for ongoing and instant support, including expertise from the three agencies accessible to healthcare providers at health facility level.
Facilitative leadership	• Collaboration aimed at enhanced advocacy and policy engagement. • Regional focal persons in the three agencies formed a functional triumvirate that facilitated collaboration because of their longstanding professional affinity and their personalities—acquainted with and respected one another's working and communication styles. • Leadership in all operational areas (technical, convening, advocacy): tasks and responsibilities divided to use available expertise and resources efficiently. • Tendency to compete was eliminated and attention focused on the common good: - Spoke with one voice and not as individual agency voices—if one agency did not participate in a discussion, the other agencies ensured that the interests of the absent agency were reflected and addressed. - Stood in for each other—took on board tasks of other agencies as needed. - Each agency contributed their available resources at a particular time and participated in activities they did not fund. - There was joint accountability for the process and no blame shifting when something went wrong.
Institutional design	Pre-existing relationship of the three agencies as UN institutions • facilitated the collaboration; • enhanced a shared understanding of the norms governing each agency and issues at hand; and • facilitated the development of a joint plan; the assignment of roles and responsibility in line with institutional mandates; and the implementation of activities in support of capacity building in countries.

ESA, Eastern and Southern Africa; CDC, Centres for Disease Control and Prevention; COVID-19, coronavirus disease (severe acute respiratory syndrome coronavirus 2 or SARS-CoV-2); ICAN, Infection Control Africa Network; IPC, infection prevention and control; MNCH, maternal, newborn and child health; MPDSR, maternal and perinatal death surveillance and response; SRHR, sexual and reproductive health and rights; (S)RMNCAH, (sexual,) reproductive, maternal, newborn, child and adolescent health; NGO, non-governmental organization; UN, United Nations; UP, University of Pretoria; WCEA, World Continuing Education Alliance.

## Lessons learned

### Start-up lessons

Commitment is a critical element in initiating and maintaining collaboration. The commitment of the ESA team inspired greater commitment from the teams of the three agencies at regional and headquarters levels to trusting and supporting this interagency collaboration at a time when there was no evidence as to the best way to ensure continuity of MNH services during the pandemic. Commitment to stepping into the unknown involved risk taking, since we did not know whether initiatives would go wrong. The collaboration facilitated regional learning and provided guidance on MNH in the context of COVID-19 while we waited for conclusive guidance from headquarters. Risk taking also took the form of pushing the boundaries, asking for help without always following the usual channels of communication and fast-tracking contracts with service providers. The focal persons were also committed to acquiring new virtual communication skills and learning to be flexible about working hours.

Collaboration between agencies, without loss of autonomy, can result in enhanced efficiency through the sharing of functions and resources. In our collaboration the three agencies largely retained their own internal structures (support, administration, and indirect costs), but provided time and management to the partnership regardless of the form. During the health emergency there was insufficient time to develop a formal collaborative framework that explicitly quantified the trade-off between shared function gains and collaboration costs. On the other hand, the pre-existing ties between the agencies made it possible to achieve good results through collaboration.

### A “united front”

Having a common goal can break down existing silos based on categories of RMNCAH. We adopted the mantra “*together each achieves more*” (TEAM) to move away from *ad hoc* joint tasks and develop a longer-term joint plan incorporating milestones of results to be achieved along the way. Our interagency collaboration also meant improved representation as a single UN body—when one agency was absent, the others ensured the issues related to the absent agency were addressed.

Through joint work, opportunities arose to link up with broader UN interagency mechanisms and there was potential to attract bigger collaborators such as the African Centers for Disease Control and Prevention (CDC).

The harmonized TEAM approach during the pandemic had the following advantages:

• A unified approach was adopted when exploring connections with country offices. This avoided fragmentation and reduced countries' transaction costs (particularly costs incurred by national government focal persons) as they only had to respond to one instead of three requests from the agencies. The UN agencies also saved time by jointly monitoring countries’ RMNCAH service continuity.
• Adopting a common position during uncertainty mitigated confusion, especially in the initial phase, and avoided potentially conflicting messages from individual agencies. This enabled a more effective exchange of best practices with a higher impact and promoted a uniform response to MNH focal persons at ministries of health who requested guidance from each agency. This coordinated and joint response proved more beneficial for planning and implementation of MNH services during a complex health emergency.
• Our joint response further reduced the competition for funds and increased available resources for the response as opposed to fragmented funding to individual agencies.
• This also assisted with leveraging additional resources from the Bill and Melinda Gates Foundation and the 2gether 4 SRHR fund.
• Each agency brought along its own stakeholders for consultation and intervention design to feed into the joint plan. Collaboration instead of independent functioning was also more appealing to target audiences. This boosted the effectiveness of program implementation, extended geographical coverage and expanded the reach for the improvement of quality of care.

### Harnessing technology to accelerate results

When the global pandemic emergency and accompanying travel restrictions set in and rendered commonly used capacity building modes like workshops and field missions impossible, innovation and the use of technology offered a solution. The joint UN–ESA offices realized that there were more participants requiring support than could be reached through webinars. A concept paper was developed which envisioned collaboration between (a) the three UN agencies to ensure customization of global guidance to the regional context, (b) educators (professional associations and councils) to develop training content and train their members, and (c) a reliable technology company to convert the training content to e-learning modules.

The World Continuing Education Alliance (WCEA) was competitively selected as the technology company for hosting the e-learning modules and disseminating the training. A major advantage of using a technology platform was cost saving. We calculated that a physical workshop would cost on average US$100,000 per workshop for about 70 participants, whereas the initial cost of setting up the modules on the technology platform was US$70,000. The program has now been running for more than two years and is reaching a much higher number of participants. Other advantages of harnessing technology include: real-time feedback and data disaggregated by demographic variables; increased reach and influence; and online and offline access to modules through downloadable documents and videos. In addition to the formal arrangement with WCEA, countries and individuals were also encouraged to explore the contents and courses available on other e-learning platforms such as the WHO Academy.

### Structures and contextual factors

Our collaboration contradicts the widely held belief that less formalized collaboration does not result in increased levels of harmonization between implementing agencies. This style of informal collaboration with a small number of important partners can be highly beneficial in instances where collaboration is required in an emergency or for a time-sensitive effort. Examining the context in which a collaborative framework operates is critical for creating an understanding of the incentives that influence agency decisions about cooperation levels and types. A common vision and agreement on shared ideas and skills can be a game changer.

Developing collaboration tools can also have an impact in times of emergency. Such tools include planning strategies for operational coordination and implementation, joint concept notes and terms of reference for specific tasks, designing communication strategies with other partners and potential funders, and utilising virtual communication, training and survey tools.

Any kind of collaboration has its challenges. In our case we faced frequent changes in global guidance early in the pandemic, necessitating reorientation of country teams and eroding trust. Competition for resources continued and the regional interagency collaboration was difficult to replicate at country level. Sustaining collaboration became challenging when key members of a country team moved away from the region. Time pressures sometimes led to delays in responding to urgent requests. Lastly, the virtual environment posed challenges for e-learning participants who had difficulty in familiarizing themselves with the app feature for accessing the e-learning modules. Discussions are ongoing to functionalize the community of practice feature of the app.

## Conclusion

There are widespread perceptions that collaborative initiatives between agencies are difficult and that they tend to yield minimum results in respect of increased efficiency or effectiveness. This collaborative effort demonstrated elements of feasibility, value addition, synergy, cost effectiveness and demonstrable results where UN agencies delivered as one. The emergency in healthcare as a ripple effect of the COVID-19 pandemic has caused agencies to rethink their collaboration models and levels of engagement within the agencies and in inter-sectoral arrangements. What has been achieved has the potential to change the direction of healthcare going forward—for the better. This approach is now being adopted in other UN regions and countries as well.

## Data Availability

The original contributions presented in the study are included in the article/**Supplementary Material**, further inquiries can be directed to the corresponding author.

## Author's Note

FG is a Maternal and Newborn Health Specialist at the UNICEF Eastern and Southern Africa Regional Office. NAK is a Medical Officer at the WHO AFRO Intercountry Support Team for East and Southern Africa. MA is a Health System Specialist at the UNFPA East and Southern Africa Regional Office.
