# Supplementary material for: In uncharted territory “together each achieves more”: a United Nations interagency collaboration for continuity of maternal and newborn health services during the coronavirus pandemic in the Eastern and Southern Africa region
Source: Front Health Serv. 2023 Aug 31;3:1230414. doi: 10.3389/frhs.2023.1230414 (PMC10501831; doi:10.3389/frhs.2023.1230414)
Supplement: Supplementary file 1 [file Datasheet1.pdf]

SUPPLEMENTARY MATERIAL

Table S1. Webinars conducted in 2020*

Date of webinar	Topic of webinar	Main responsibility	Number of attendants	Remarks
9 April 2020	Sharing regional guidance on the continuity of essential maternal and neonatal services	UNICEF	85	25 countries participated in the webinar, of which a few were outside the ESA region (Sierra Leone, Palestine, Jordan, Egypt, Libia)
23 April 2020	Experience sharing by countries based on the phases of COVID:<br>· Ethiopia: coordination mechanism<br>· South Africa: development of country-specific guidelines	UNFPA	120	Participating countries were asked about<br>· COVID phase they were in<br>· whether continuity of MNH services was being monitored and by what data source
7 May 2020	Country experience sharing continued:<br>· South Africa: M&E of COVID in pregnancy<br>· Zimbabwe: virtual health-facility rapid assessment for monitoring of continuity of MNH services during COVID<br>· Uganda: M&E using DHIS2 for trends in continuity of MNH services	UNFPA	149	Participating countries were asked in a poll what topic they would like to include in the next webinar
21 May 2020	COVID-19 newborn care:<br>· In health care facilities and postnatal care<br>· Intrapartum care for suspected and confirmed COVID cases	UNICEF/WHO	145	National guidelines developed at that time in 4 countries in Africa shared with participants

Date of webinar	Topic of webinar	Main responsibility	Number of attendants	Remarks
4 June 2020	· Implications of COVID-19 for maternal and newborn health (LiST projections)<br>· Key considerations for postpartum care in the light of COVID<br>· Postpartum family planning within the COVID context	WHO	127	Discussion on taking the zoom webinars to a higher level through collaboration with a tech company<br>Concept note on e-learning presented<br>Next steps for collaboration with key RMNCAH stakeholders in ESA Region discussed – use of technology for<br>sustainable and resilient capacity building through continuous professional development (CPD) and lifelong learning<br>achieving better healthcare outcomes for mothers and their newborns
18 June 2020	Community maternal and newborn health services	WHO	106	
2 July 2020	Infection Prevention and Control (IPC) Part 1: Considerations applicable to maternity setting in the context of COVID-19<br>· Personal protective equipment (PPE)<br>· Hand and respiratory hygiene	UNICEF/WHO	164	The video recording of the IPC training was edited and posted on the three agencies' websites. Following example taken from UNFPA website: https://esaro.unfpa.org/en/video/infection-prevention-and-control-maternity-settings-within-covid-19-context-part-1
9 July 2020	IPC training Part 2: Deep dive into key IPC considerations for inpatient settings: maternity wards, labour/delivery wards, and surgical theatres	UNICEF/WHO	112	Information on PPE much appreciated by participants – increased country engagement (e.g. request for more information and for budget for PPE)
23 July 2020	IPC training Part 3: IPC for community delivery	UNICEF/WHO	124	

Date of webinar	Topic of webinar	Main responsibility	Number of attendants	Remarks
6 August 2020	MPDSR Part 1: MPDSR and implications of COVID<br>Assigning cause of death during pregnancy, childbirth and puerperium, including examples of COVID-19 as cause of death	WHO	135	At this point in time, most countries in ESA Region had put mechanisms in place to respond to COVID-19. That is why the webinar topics changed from studying COVID alone to exploring the implications of COVID in the health system as applicable to MPDSR, the health information system, etc.
20 August 2020	MPDSR Part 2: Sustaining a quality MPDSR system, including accurate cause of death classification coding during COVID-19	WHO	116	
3 September 2020	MPDSR Part 3: Community MPDSR<br>Experience of Bangladesh	UNFPA	124	
18 September e2020	Standards for improving quality of care for small and sick newborns at health facility level	UNICEF/WHO	104	
1 October 2020	Gender-based violence (GBV) in the context of COVID:<br>Survivor-centred approach to clinical management of rape survivors<br>Joint program experience on multi-sectoral response to VAWG<br>Country experience on mobile GBV one-stop centre	UNFPA	96	
15 October 2020	Organizational approach to implementation of Quality of Care (QoC)	UNICEF	134	Last webinar before migration to the WCEA app

Date of webinar	Topic of webinar	Main responsibility	Number of attendants	Remarks

COVID-19, coronavirus disease (severe acute respiratory syndrome coronavirus 2 or SARS-CoV-2); CPD, continuous professional development; DHIS2: district health information software, version 2; ESA, Eastern and Southern Africa; GBV, gender-based violence; IPC, infection prevention and control; LiST, Lives Saved Tool; M&E, monitoring and evaluation; MNH, maternal and newborn health; MPDSR, maternal and perinatal surveillance and response; PPE, personal protective equipment; RMNCAH, reproductive, maternal, newborn, child and adolescent health; VAWG, violence against women and girls; WCEA, World Continuing Education Alliance.

Allocation of responsibilities	
**Agency**	**No. of webinars**
UNFPA	4
WHO	4
UNICEF	2
UNICEF/WHO	5

Number of participants	
**TOTAL**	**1841**
Median	124
Mean	123
Min	85
Max	164

* More information and links to presentations are available at: https://www.healthynewbornnetwork.org/event/joint-regional-webinar-series-monitoring-of-continuity-of-mnh-services-africa/

Table S2. MNH modules on the e-learning portal*

MNH area	Module topic	URL
**WHO Labour Care Guide**	1. Introduction to the WHO Labour Care Guide	English: https://rise.articulate.com/share/Mh14kys1UvG2R7yfFYI1dCzX3Ka28XbP<br>French: https://rise.articulate.com/share/jBG2PTcFz3xxmmbDPoZfXQHEh-0UQWRL<br>Portuguese: https://rise.articulate.com/share/pJl-G47pcW8n-st0gotAk-IpbHosyXjA
**Maternal Death Surveillance and Response (MDSR)**	2. Key definition of maternal death surveillance	English: https://rise.articulate.com/share/_rEtqDq_MnuQdYEa-XkrXib_RqBvVZCi#/<br>French: https://rise.articulate.com/share/fLQjRkN3dr_nya1L0bdKXbG8U05R_R7g<br>Portuguese: https://rise.articulate.com/share/aG1wL0MGVM51qc_-LZH4FPD8kBl9j6d2
	3. Introduction to medical certification of cause of death (MCCoD)	https://rise.articulate.com/share/QTn3INIhIYXfiE1k-RGH9v69VtzxFYeH
	4. The WHO application of ICD-10 to deaths during pregnancy, childbirth, and the puerperium: ICD-MM	English: https://rise.articulate.com/share/fc6UU3r44uVKK4CunCxJCz18mW8u8jAy<br>French: https://rise.articulate.com/share/X_U5eIyPt48XpwHiuaXQdb8W-rPeiBb3<br>Portuguese: https://rise.articulate.com/share/S6npwp6oQfPb3nnnjfdIqOURG5Cnqo7k
	5. Monitoring and evaluation of maternal and perinatal death surveillance and response system	English: https://rise.articulate.com/share/OtegcshzxsbNkWFOh64WfI3unYUOfK5-<br>French: https://rise.articulate.com/share/PpbtKsQHzJIkR41peIw94LPn8EpZeTxO<br>Portuguese: https://rise.articulate.com/share/7BduSVyIk6AxoHYJjamtaAPZg6pBTUGI
**Perinatal Death Surveillance and Response (PDSR)**	6. Key definitions, identification and reporting of perinatal deaths	https://rise.articulate.com/share/aFx-csczH5bhYmnW5DMJWAZ5Dd5cNtxs#/
	7. Collecting, analysing and documenting perinatal data	https://rise.articulate.com/share/YA9lcQ_GpWFxmZ-oyDFPRlRnJ7akwyNe#/
	8. Perinatal mortality review principles and common myths and misconceptions	https://rise.articulate.com/share/EnauieN9BDXj50Av9sJzTP_29dWppICs
	9. Recommending and implementing solutions	https://rise.articulate.com/share/UXFiwAWi6sq9d5bXDC0o1WpfhmqKu5RP

* The modules are available at the following website/app: https://wcea.education/. They can be taken for CPD points (click on 'Login to LMS') or for personal development without earning CPD points (click on 'Webinars').
